# Letter from E. V. Stoddard, Berlin

**Published:** 1879-07

**Authors:** E. V. Stoddard

**Affiliations:** Berlin


### Correspondence.

---

BERLIN, June 1st, 1879.

MY DEAR DOCTOR :—The many engrossing experiences, in the lands of ruins and historic interest, must be my apology for not sooner complying with your parting request that I should write to you. Here, I am a fixture for the present, and so have begun to adjust my internal conditions (mentally) to the circumstances of my environment, as Herbert Spencer would say. I hardly know what to write you of, so much have I met of great interest to myself. I early went to Rome, after landing in Liverpool last March, and spent nearly a month there under the bright skies, and in the warm sunshine of Italy, and found it a most welcome substitute for the cold, gray skies of England, and the, then, cold atmosphere of Paris. No place in the world offers, to the visitor, the endless field of interest which Rome presents. One can revel, at will, amid the relics of a historic past, or luxuriate amid the treasures of art, until his æsthetic nature is so filled with enjoyment of the beautiful that he almost sighs for some of the sterner duties of life to come to his aid and relief. So we found it, and, reluctantly, left the Eternal City for a short stay in Florence, Venice, etc. In Rome I looked the hospitals over pretty thoroughly, and can only say they are good, bad and indifferent. The best is the large Surgical Hospital of "San Geacomo in Augusta." This, with a capacity of 1,200 beds, is one of the finest Hospitals I have visited. It is supplied with all modern appliances, and is scrupulously *clean*, which is not the case with the Italian Hospitals generally.

The sewer system of ancient as well as modern Rome was most interesting to me. I explored the "Cloaea Maxima," and in doing so, was very profoundly impressed with the fact that those old Romans "*built better than they knew*," for this old sewer, constructed nearly 2,000 years ago, is doing its duty to-day as well and faithfully as when first constructed, and will continue to do so for a thousand years to come despite the destructive influences of "the gnawing tooth of time." Florence and Venice we enjoyed most fully, especially in their Art treasures. At Florence the

Anatomical Museum is the largest and most interesting I have seen. The collection of wax models of muscular, nervous, vascular and osseous tissue is wonderfully beautiful and perfect. Susini's best work is here. From Venice we came on to Munich, stopping a day at Trent to break the journey. This place, where the old Councils of Trent were held in 1545 and 1563 was quite interesting. It is beautifully situated in the Austrian Tyrol among the Alps.

At Munich, a longer stay opened much of interest. The University is excellent in its arrangements and appointments. I was much pleased with my experiences with Prof. Ziemssen. He was most cordial and polite, and interested me much in studies he was engaged in making, in the peristallic movements of the intestines, and the contractions of the stomach under the stimulus of the direct or induced currents. His deductions are interesting and are borne out by the vivisections he is making. He appreciates American fondness for study, and, like most of the German Professors I have met, feels that a strong bond of sympathy exists between German and American Scholars. Both his and Prof. Nussbaum's clinics are supplied with every appliance conducive to illustration and instruction. Prof. Bauer was exceedingly pleasant. Munich is very attractive to the student. Its Art treasures are numerous, and most systematically and scientifically arranged. On the whole, I consider it an admirable place for study. From Munich we went to Leipsic. Here I enjoyed the days very fully in the attractions of the University. Prof. Ludwig, of Physiology, is a thorough German, and student. He is full of enthusiasm, and his Laborasory is most interesting. I passed a delightful afternoon with him in it, listening to his deductions from his studies on the constitution of the blood, muscular contractility and nervous irritability. His apparatus is very delicate.

Prof. Kolbe was exceedingly kind; he seemed to take much pleasure in inducting me into the mysteries of his extensive and admirably appointed Laboratory. He is most interesting, and enjoys sharing the rich accumulations of his study and observation with others. Knowing his views upon the physiological action of Salicylic Acid, I took pains to become acquainted with his opinions from personal statements. He has written somewhat upon the subject, and I have been much interested in reading a published article of which he gave me a copy. It is so interesting in some of its conclusions that I have translated and enclosed a portion of it.* I think it will interest the readers of the *Journal*, coming from one who occupies so prominent a position in the world of science as does Prof. Kolbe. With Prof. Hofmann (Prof. of Hygiene). I visited the "Municipal Hospital." This was constructed a few years ago under the direction of Wunderlich and Thiersch, and is an admirable institution in its construction and appointments. It has a capacity of five hundred beds and is well conducted.

Leipsic with its attractions had to be left; and, accordingly, we came here some ten days since. I expect to remain here until June 15th, when we go to Paris for a month, before going to England. The University here offers many attractions, and I shall feel loth to leave Berlin. Prof. Virchow recently addressed the Medical Society of Berlin upon the subject of the Astrakan Plague. He dwelt upon the fact that our knowledge of the physiological characteristics of the disease is very slight, and was severe in his strictures upon the Russian Government for failing to send out competent men to observe its character. He thought a blockade of the Russian frontier very deceptive. He questioned the value of Sulphurous Acid as a disinfectant, because he thought it doubtful whether this agent is able to penetrate clothing in such a way as to destroy the germs. He recommended as a better plan, that all infected articles be placed in a chamber, raised to a temperature of 120° Centigrade by means of steam pipes.

But I shall weary you, I fear. I will try to write you again from Paris, when I will give you some facts relative to sewer systems of the great cities I have visited, etc.

My kindest remembrances to your family. To all my colleagues in Buffalo please extend my kindest regards. I look forward with pleasure to the time when our mutual work begins.

I am, as ever,

Very sincerely yours,

E. V. STODDARD.

*This translation will appear in our next number.

